# Acute Cerebillitis Due to Salmonella typhimurium Infection in an Adult: A Report of an Unusual Case

**DOI:** 10.7759/cureus.54181

**Published:** 2024-02-14

**Authors:** Govind Shiddapur, Mohith Prakash Kondapalli, Vutukuru Kalyan Kumar Reddy, Saimounika Adapa, Heer Shah

**Affiliations:** 1 Department of General Medicine, Dr. D. Y. (Dnyandeo Yashwantrao) Patil College, Hospital and Research Centre, Dr. D. Y. Patil Vidyapeeth, Pune, IND; 2 Department of Microbiology, Dr. D. Y. (Dnyandeo Yashwantrao) Patil College, Hospital and Research Centre, Dr. D. Y. Patil Vidyapeeth, Pune, IND

**Keywords:** impaired cerebellar function, inflammatory illness, salmonella typhimurium, enteric fever, cerebillitis

## Abstract

Acute cerebellitis is an inflammatory illness that may manifest as a primary, para-infectious, or post-infectious disease. The clinical manifestations of acute cerebellitis are traditionally characterized by fever, vomiting, headache, and altered sensorium, accompanied by impaired cerebellar function corroborated by neuroradiography alterations. Acute cerebellitis may lead to a potentially fatal increase in pressure within the skull, requiring immediate and critical neurosurgical surgery. It is important to note that cerebellar symptoms may not be evident initially. This report provides a comprehensive analysis of a case of a 57-year-old male patient who had been diagnosed with acute cerebellitis caused by an infection with *Salmonella typhimurium*.

## Introduction

Acute cerebellitis is an uncommon inflammatory condition defined by the sudden onset of cerebellar impairment, sometimes without an identifiable etiology. The term "acute cerebellar ataxia" is commonly linked to a condition characterized by impaired functioning of the cerebellum. Nevertheless, there is a notable convergence in its application due to the ambiguous mechanisms of the fundamental pathology [1].

The documented infectious agents that can either cause or be linked to acute cerebellitis include varicella infection, mumps, measles, rubella, Epstein-Barr virus, cytomegalovirus, herpes simplex virus, influenza virus, parainfluenza virus, poliovirus, coxsackie virus, and bacterial infections such as Salmonella typhi, *Borrelia burgdorferi*, *Coxiella burnetii,* *Bordetella pertussis*, and *Mycoplasma pneumoniae* [2]. Acute cerebellitis can occasionally occur in the absence of any indications of a prior or concurrent infection [3].

The usual course of the condition is frequently mild, characterized by signs of impaired cerebellar function. The clinical manifestation comprises either moderate or severe pyrexia, cephalalgia, alterations in cognitive function, and the emergence of cerebellar manifestations such as ataxia, dysarthria, tremors, nystagmus, and hypotonia [4]. However, acute cerebellitis can occasionally present a substantial life-threatening danger due to its potential to cause intracranial hypertension, a well-documented outcome. Possible consequences include tonsillar herniation, hydrocephalus, and severe atrophy of the cerebellum. Most recorded cases have occurred in children, while instances in adults have been extremely rare.

## Case presentation

A 57-year-old male patient arrived at our medical center with symptoms of dizziness, vomiting, and inability to walk that had persisted for the past two days. The patient complained of dizziness such that his surroundings were revolving around him, which was associated with nausea and five episodes of vomiting that were not projectile, bilious, or blood-tinged, and associated with food particles. The patient was unable to walk for small distances and was swaying to the left, and he needed his son's support for his daily activities. The patient was known to be diabetic and hypertensive and had been on daily medications (tablet metformin 500 mg twice a day, tablet telmisartan 20 mg twice a day) for the last 15 years. The patient also had a history of fever seven days prior to presentation, associated with three episodes of loose stools. The local hospital had treated him symptomatically, and he experienced relief after two days.

The patient's physical examination indicated that he had a typical physique and were adequately nourished. The patient exhibited a normal body temperature, with a pulse rate of 118 beats per minute, blood pressure of 140/100 mmHg, 98% oxygen saturation level, and a rate of respiration of 20 cycles per minute.

During the systemic examination of the central nervous system (CNS), the patient was conscious, oriented, and alert. Hypotonia was present on the left upper limb (UL) and lower limb (LL). On further examination, we noticed swaying to the left side. On further examination, the patient exhibited no nystagmus but was unable to perform the finger-nose-finger test on the left UL. We noted dysdiadochokinesia on the left UL, observed a positive rebound phenomenon on the left hand, and found a positive pendular knee jerk on the left LL. The patient was unable to complete the foot-tapping test on the left LL. The bilateral flexor-plantar reflex was noted.

Routine blood tests came back normal except for the leucocytosis (Table 1).

**Table 1:** Routine blood investigations TLC: total leucocyte count; SGOT: serum glutamic-oxaloacetic transaminase; SGPT: serum glutamic pyruvic transaminase; RBS: random blood sugar; HIV: human immunodeficiency virus; HBsAg: hepatitis B antigen; HCV: hepatitis C virus; NR: non-reactive: HbA1c : glycated haemoglobin

Parameters (normal limit)	Day 1	Day 4	Day 8	Day 12	Day 16
Hemoglobin (12-16 gm/dl)	13.3	12.8	12.5	12.3	12.9
TLC (4,000-10,000 /µL)	16700	12300	10300	8900	8500
Platelets (1,50,000-4,10,000 /µL)	333000	345000	350000	356000	327000
Serum bilirubin (0.2–1.2 mg/dL)	0.89	1.0	0.78	1.2	1.1
SGOT (8–48 IU/L)	40	50	43	46	41
SGPT (7–55 IU/L)	48	34	34	35	33
Serum urea (17–49 mg/dL)	34	48	34	40	38
Serum creatinine (0.6–1.35 mg/dL)	0.59	0.88	0.78	0.89	0.87
Serum sodium (135-145 mmol/Lt)	136	138	140	134	136
Serum potassium (3.5-5.1 mmol/Lt)	3.7	3.5	3.6	4.0	3.9
RBS (up to 140mg/dl)	130	134	140	135	139
HbA1c	6.1				
HIV/HBsAg/HCV	NR				
Serum calcium (8.6-10.2 mg/dl)	8.4				
Serum magnesium (1.8-2.40 mg/dl)	2				
Serum phosphorous (2.8-4.5 mg/dl)	3.60				
Serum ammonia (20-120 mcg/dl)	53				

**Figure 1:**
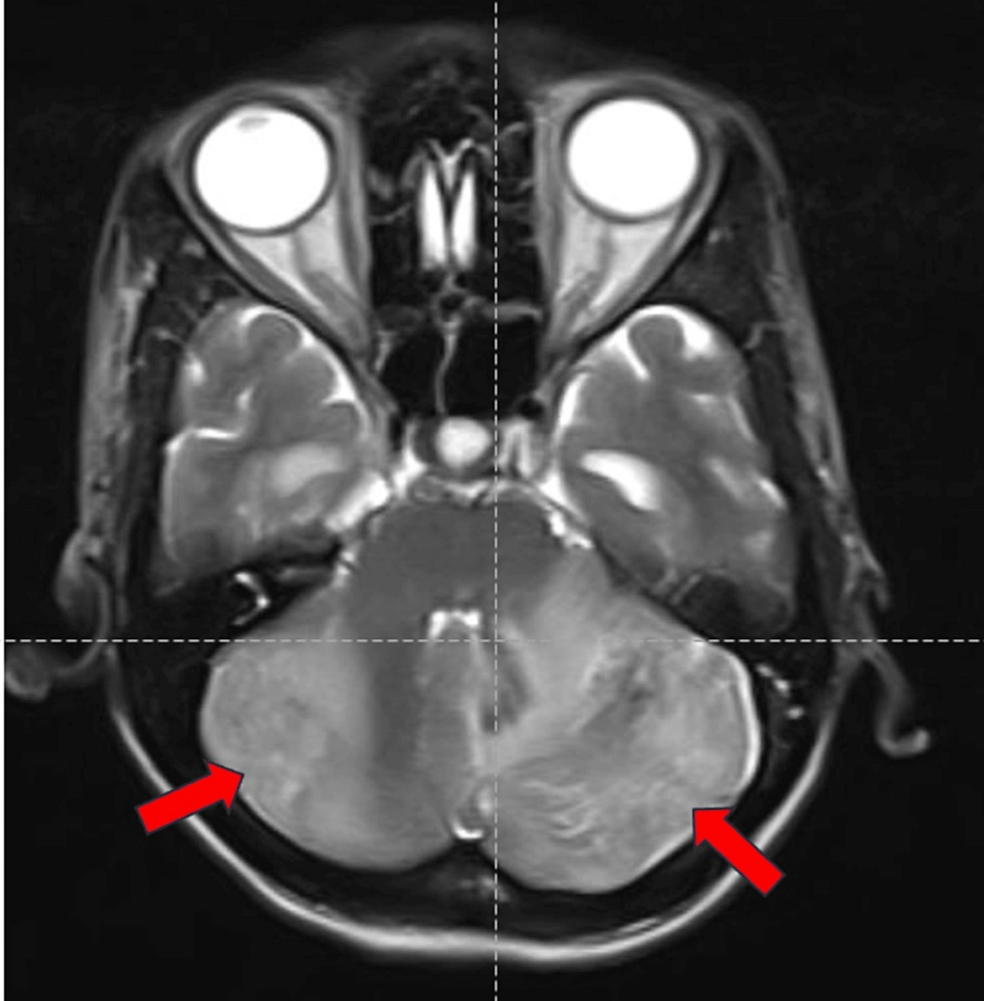
MRI of the brain (axial section) shows a few ill-defined hyperintense lesions in the bilateral cerebellar hemispheres involving both gray and white matter on the T2 image shown by the red arrows.

**Figure 2:**
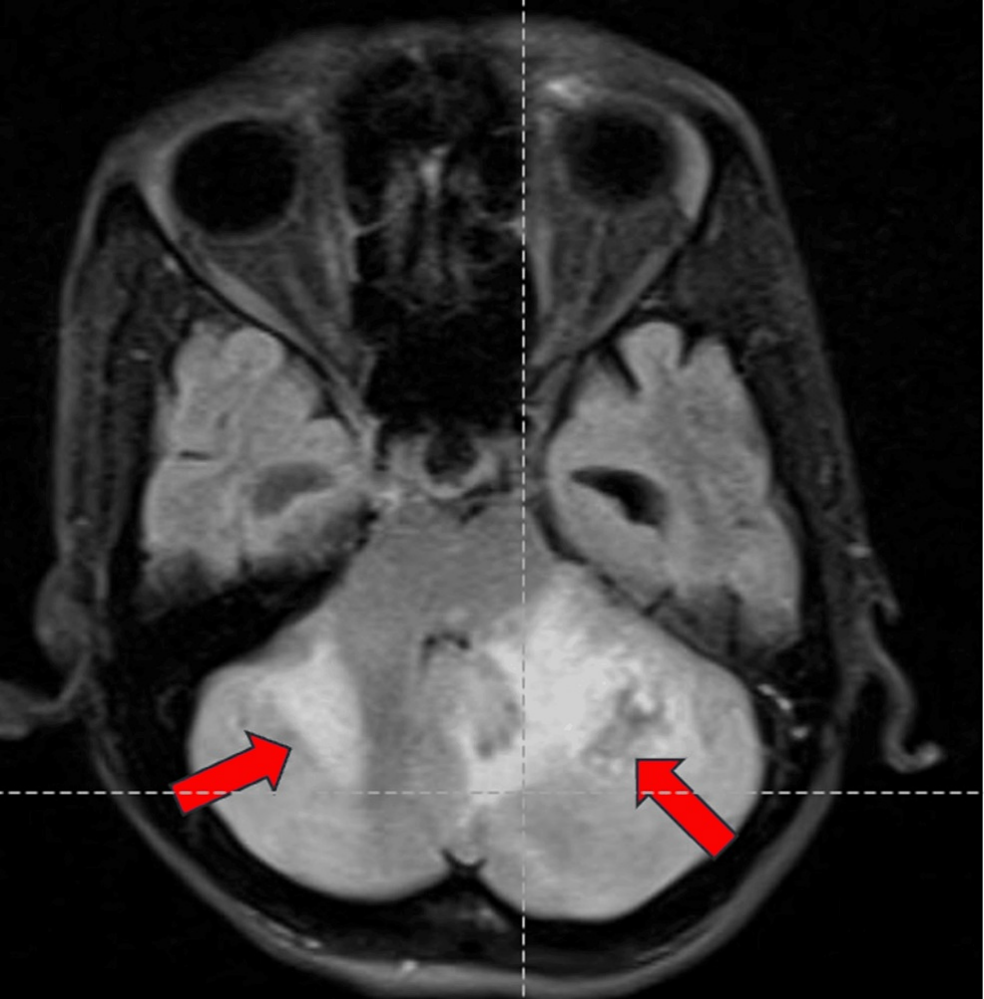
MRI of the brain (axial section) with a fluid-attenuated inversion recovery imaging sequence showing few ill-defined hyperintense lesions are noted in bilateral cerebellar hemispheres involving both gray and white matter, shown by red arrows.

**Figure 3:**
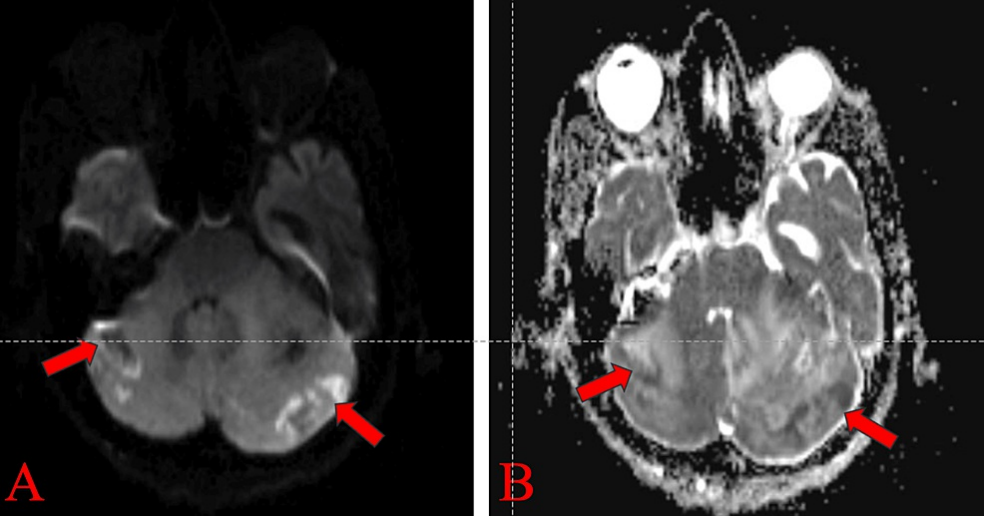
MRI of the brain (axial section) with diffusion-weighted imaging sequence showing few ill-defined hyperintense lesions are noted in bilateral cerebellar hemispheres involving both gray and white matter (A) with corresponding low apparent coefficient value (B), shown by red arrows.

Magnetic resonance imaging (MRI) of the brain showed a few ill-defined hyperintense lesions in the bilateral cerebellar hemispheres involving both gray and white matter, suggestive of acute cerebellitis (Figures 1-3).

On further evaluation, as the patient had a previous history of fever, common causes of cerebellitis were identified, which revealed a widal test reaction (1:320) (Table 2).

**Table 2:** Additional investigations NS1: nonstructural protein 1; IgM: immunoglobulin M; IgG: immunoglobulin G

Test	Results
Dengue NS1 antigen	Non-reactive
Anti-dengue IgG	Non-reactive
Anti-dengue IgM	Non-reactive
Rapid malaria test	Non-reactive
Widal test	Reactive (1:320)
Cytomegalo virus IgM	Non-reactive
Cytomegalo virus IgG	Non-reactive
Epstein Barr virus IgM	Non-reactive
Epstein Barr virus IgG	Non-reactive
Varicella zoster virus IgM	Non-reactive
Varicella zoster virus IgG	Non-reactive

**Figure 4:**
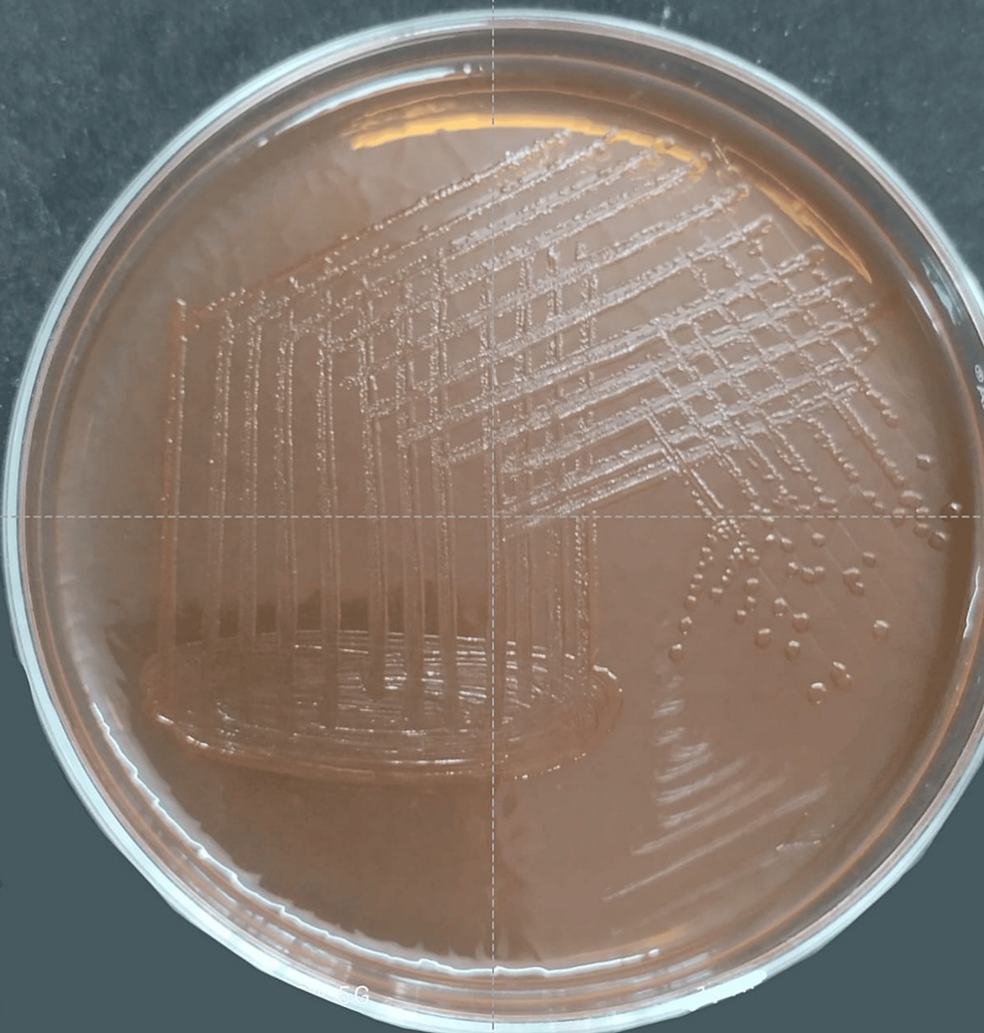
Colorless, non-fermenting colonies of 2–3 mm in size were seen after 24 hours of incubation at 37°C on MacConkey Agar. Colonies were round with a smooth, low-convex surface and irregular edges suggestive of Salmonella typhimurium.

A blood culture was sent for further confirmation, which revealed colorless, non-fermenting colonies of 2-3 mm in size after 24 hours of incubation at 37°C on MacConkey agar. Colonies were round with a smooth, low-convex surface, and irregular edges suggestive of *Salmonella Typhimurium* (Figure 4).

Based on these reports and investigations, the medical team diagnosed the patient with acute cerebillitis caused by post-infectious *S. typhimurium*. Treatment was initiated based on the diagnosis. Injection of ceftriaxone, 2 gm, intravenously twice a day, was given for 14 days, and injection of dexamethasone, 8 mg, intravenously thrice a day for three days, followed by 8 mg, intravenously twice a day for three days, and tapered down to 8 mg, intravenously once a day for three days. After receiving regular physiotherapy and completing a course of antibiotics, the patient experienced a significant improvement in their quality of life and was discharged after 16 days. Follow-up wasuneventful.

## Discussion

Acute cerebellitis is an inflammatory illness that results a temporary impairment of cerebellar function. Westphal and Batten described it for the first time in 1872. In 2007, the International Multiple Sclerosis Study Group officially categorized cerebellitis as a separate and independent disorder. It can present itself as a primary infection, a condition that occurs after an infection, or a condition that follows immunization [5]. The main clinical symptoms of acute cerebillitis include headache, vomiting, lethargy, changes in consciousness, coma, ataxia, and fever. Acute cerebellitis is a clinical disease of unknown etiology, which may be caused by viral or bacterial infections.

Westphal first recorded the presence of cerebellar ataxia in cases of enteric illness in 1872. Verifying the diagnosis of enteric fever involved isolating the bacteria in blood culture, observing a significantly positive widal test, and detecting an elevation in antibody levels. Most of these patients showed long-lasting disorientation, myelopathy, or extrapyramidal involvement. The specific mechanism behind the development of cerebellar ataxia in enteric fever is yet to be identified. The neurological consequences of enteric fever, such as brain edema and hemorrhages, have been linked to metabolic disruptions, blood poisoning, a very high temperature, and non-specific alterations in the brain [6,7].

To diagnose acute cerebellitis, healthcare providers must perform a detailed evaluation of the patient's medical history and conduct a thorough examination of their overall and neurological state. There are no convincing diagnostic markers in laboratory tests. Consideration of acute cerebellitis should be made after a thorough evaluation of potential alternative diagnoses, including toxic exposure, infections, and structural anomalies. The MRI in patients with acute cerebellitis indicates the cerebellum can be affected in several ways, but the most frequent presentation is the presence of diffuse abnormalities in both hemispheres [8].

There may be widespread edema in the outer layer of the brain. Interestingly, in some cases, the outcomes of the MRI scan seem to fall within the anticipated range. Nevertheless, none of the MRI findings are specifically indicative of acute cerebellitis. However, in cases where a patient has unevenly localized impairments and/or changes in mental awareness, it is imperative to utilize MRI imaging [9].

There is currently no universally agreed-upon agreement about the management of cerebellitis and the available therapy options. In clinical practice, patients suffering from acute cerebellitis have been administered steroids, intravenous immunoglobulin, and antiviral medications as part of their treatment [10]. Given the potential correlation between ataxia and viral encephalitis as well as bacterial meningitis, it is recommended to explore the use of antimicrobial therapy [11,12]. The use of steroids remains a topic of debate, with no prevailing agreement [13,14].

In their trial, Kornreich et al. administered steroids as the initial treatment for people with acute cerebellitis [15]. In addition, antibiotics were administered to seven persons, and intravenous immunoglobulin was also administered to four patients.

The case report authored by Yiş et al. detailed the management of an eight-year-old female patient who presented with symptoms of headaches, dizziness, nausea, and vomiting [15]. Dexamethasone was provided to the patient at their department. The authors of the cited case report suggested that, for moderate cases, using normal dexamethasone medication instead of high-pulse methylprednisolone treatment could be a good therapeutic option. In cases of severe hydrocephalus in acute cerebellitis, it is recommended to immediately undergo neurosurgical treatments, such as external ventricular drainage, ventricular peritoneal shunt, and posterior fossa decompression [16,17].

## Conclusions

Acute cerebellitis may lead to a dangerous increase in pressure within the skull, known as increased intracranial pressure. In certain cases, immediate neurosurgical surgery may be necessary to save the person's life. It is important to note that cerebellar symptoms may not be evident initially. Neuroimaging plays a vital role in supporting the diagnosis, identifying severity, and directing the therapy strategy. In the current case, the diagnosis of enteric fever was verified with a positive blood culture, a very positive widal test, and increasing antibody titers.

## References

[1] Patel P, Rayamajhi S, Tokala H, Laird-Fick H (2013). An unusual cause of altered mental status in elderly-acute cerebellitis: a case report and review. Case Rep Med.

[2] Lee KY, Lee KS (2014). Elevated cerebrospinal fluid IgG index in acute cerebellitis presenting with sudden onset headache. Neurol Asia.

[3] Hayase Y, Tobita K (1997). Probable post-influenza cerebellitis. Intern Med.

[4] Sawaishi Y, Takada G (2002). Acute cerebellitis. Cerebellum.

[5] Krupp LB, Banwell B, Tenembaum S (2007). Consensus definitions proposed for pediatric multiple sclerosis and related disorders. Neurology.

[6] Scragg J, Rubidge C, Wallace HL (1969). Typhoid fever in African and Indian children in Durban. Arch Dis Child.

[7] Ramachandran S, Wickremesinghe HR, Perera MV (1975). Acute disseminated encephalomyelitis in typhoid fever. Br Med J.

[8] De Bruecker Y, Claus F, Demaerel P (2004). MRI findings in acute cerebellitis. Eur Radiol.

[9] Goenka A, Michael BD, Ledger E (2014). Neurological manifestations of influenza infection in children and adults: results of a national British surveillance study. Clin Infect Dis.

[10] Go T (2003). Intravenous immunoglobulin therapy for acute cerebellar ataxia. Acta Paediatr.

[11] Schwartz JF (1972). Ataxia in bacterial meningitis. Neurology.

[12] Bodegas I, Martínez-Bermejo A, de Miguel MJ, López-Martín V, de José MI, García-Hortelano J (1998). Brain stem encephalitis in childhood [Article in Spanish]. Rev Neurol.

[13] Göhlich-Ratmann G, Wallot M, Baethmann M (1998). Acute cerebellitis with near-fatal cerebellar swelling and benign outcome under conservative treatment with high dose steroids,. Eur J Paediatr Neurol.

[14] Kornreich L, Shkalim-Zemer V, Levinsky Y, Abdallah W, Ganelin-Cohen E, Straussberg R (2016). Acute cerebellitis in children: a many-faceted disease. J Child Neurol.

[15] Yiş U, Kurul SH, Cakmakçi H, Dirik E (2008). Acute cerebellitis with cerebellar swelling successfully treated with standard dexamethasone treatment. Cerebellum.

[16] Waqas M, Hadi YB, Sheikh S, Shamim SM (2016). Acute cerebellitis successfully managed with temporary cerebrospinal fluid diversion using a long tunnel external ventricular drain: a long-term radiological follow-up of two cases. BMJ Case Rep.

[17] Hacohen Y, Niotakis G, Aujla A (2011). Acute life threatening cerebellitis presenting with no apparent cerebellar signs. Clin Neurol Neurosurg.

